# IDSSIM: an lncRNA functional similarity calculation model based on an improved disease semantic similarity method

**DOI:** 10.1186/s12859-020-03699-9

**Published:** 2020-07-31

**Authors:** Wenwen Fan, Junliang Shang, Feng Li, Yan Sun, Shasha Yuan, Jin-Xing Liu

**Affiliations:** School of Information Science and Engineering, Qufu Normal University, Rizhao, 276826 China

**Keywords:** LncRNA functional similarity, Disease semantic similarity, lncRNA-disease associations

## Abstract

**Background:**

It has been widely accepted that long non-coding RNAs (lncRNAs) play important roles in the development and progression of human diseases. Many association prediction models have been proposed for predicting lncRNA functions and identifying potential lncRNA-disease associations. Nevertheless, among them, little effort has been attempted to measure lncRNA functional similarity, which is an essential part of association prediction models.

**Results:**

In this study, we presented an lncRNA functional similarity calculation model, IDSSIM for short, based on an improved disease semantic similarity method, highlight of which is the introduction of information content contribution factor into the semantic value calculation to take into account both the hierarchical structures of disease directed acyclic graphs and the disease specificities. IDSSIM and three state-of-the-art models, i.e., LNCSIM1, LNCSIM2, and ILNCSIM, were evaluated by applying their disease semantic similarity matrices and the lncRNA functional similarity matrices, as well as corresponding matrices of human lncRNA-disease associations coming from either lncRNADisease database or MNDR database, into an association prediction method WKNKN for lncRNA-disease association prediction. In addition, case studies of breast cancer and adenocarcinoma were also performed to validate the effectiveness of IDSSIM.

**Conclusions:**

Results demonstrated that in terms of ROC curves and AUC values, IDSSIM is superior to compared models, and can improve accuracy of disease semantic similarity effectively, leading to increase the association prediction ability of the IDSSIM-WKNKN model; in terms of case studies, most of potential disease-associated lncRNAs predicted by IDSSIM can be confirmed by databases and literatures, implying that IDSSIM can serve as a promising tool for predicting lncRNA functions, identifying potential lncRNA-disease associations, and pre-screening candidate lncRNAs to perform biological experiments. The IDSSIM code, all experimental data and prediction results are available online at https://github.com/CDMB-lab/IDSSIM.

## Background

Genome sequence analysis has shown that only less than 2% of human genome sequence can encode protein, that is, about 20,000 protein-coding genes, and more than 98% of human genome sequence do not encode protein, yielding a great number of non-coding RNAs (ncRNAs) [1–3]. In fact, it has been widely acknowledged that ncRNAs also play a key regulatory role in various biological processes [4, 5]. As a member of ncRNA family, long non-coding RNAs (lncRNAs) defined as ncRNAs with more than 200 nucleotides in length have been suggested as potential drivers of several diseases more recently [4, 6]. For instance, Gregory et al. reported that lncRNA HOTAIR promotes proliferation, survival, invasion, metastasis, and drug resistance in lung cancer cells [7]. Wang et al. summarized several lncRNAs that have been reported to be involved in the pathogenesis of Alzheimer’s disease, Parkinson’s disease, Huntington’s disease, and amyotrophic lateral sclerosis [8]. Therefore, inferring lncRNA functions, as well as detecting lncRNA-disease associations, can help us to deeply understand the pathogenesis of human diseases [9, 10]. For inferring lncRNA functions, a simple but efficient way is to develop functional similarity calculation model that inferring lncRNA-lncRNA functional similarities using their known functions and associations with specific diseases. Compared with biological experiments, the functional similarity calculation model is a valuable supplement to characterize lncRNA functions with less time and costs, which can be further studied by lncRNA-disease association detection methods to better understand underlying genetic mechanisms of human diseases at lncRNA level, leading to more accurate associations between lncRNAs and diseases being captured [11–13].

Many lncRNA functional similarity calculation models have been proposed so far [12–16], which mainly fall into four categories [17]. The first is based on the lncRNA expression profile. Since the lncRNA expression profile can characterize details of lncRNA in digital form, expression similarity between two lncRNAs can be calculated using correlation measures, which have strong link to functional similarity. Chen et al. proposed LRLSLDA method to predict lncRNA-disease associations, where Spearman correlation coefficient was used to measure expression similarity between expression profiles of each lncRNA pair, which was combined with lncRNA Gaussian interaction profile kernel similarity to obtain the lncRNA functional similarity [14]. The second is based on the gene ontology (GO) terms since many lncRNAs have been annotated with GO terms, which are broadly adopted for biological functional descriptions. Yu et al. utilized a Bayesian prior probability strategy, as well as associations between lncRNAs and GO terms, to measure the lncRNA functional similarity [15]. The third is based on lncRNA interactions with other biomolecules. It has been believed that lncRNAs normally interacting with other biomolecules, such as miRNA and mRNA, in a complicated way to jointly affect diseases. Therefore, measuring the lncRNA functional similarity through its interactions with other biomolecules is reasonable. Cheng et al. developed IntNetLncSim model to calculate the lncRNA functional similarity based on the integration of two interaction networks (mRNA-mRNA, miRNA-mRNA) and the lncRNA-regulatory network [12]. The fourth is based on the lncRNA-disease associations. Assuming that similar lncRNAs may show similar functions, and therefore affect similar diseases, the lncRNA functional similarity can be measured using lncRNA-disease associations and disease semantic similarity. Chen et al. proposed both LNCSIM1 and LNCSIM2 models to measure the lncRNA functional similarity, the former based on directed acyclic graphs (DAGs) and the later based on the information content (IC) to calculate the disease semantic similarity [16]. Their reliable performance improvements have been demonstrated in both cross validation and case studies. Nevertheless, they also have several limitations need to be addressed. For example, semantic contributions of different disease terms at the same layer cannot be effectively distinguished in LNCSIM1 and the accuracy of IC value always depends on the information integrity of DAGs in LNCSIM2. Huang et al. therefore developed an edge-based calculation model ILNCSIM to measure the lncRNA functional similarity, main improvement of which comes from the combination of the concept of IC and the hierarchical structure of DAGs for calculating disease semantic similarity [13].

In this study, inspired by previous models, especially LNCSIM1, LNCSIM2 and ILNCSIM, we presented an lncRNA functional similarity calculation model, IDSSIM for short, based on an improved disease semantic similarity method. Highlight of the improved disease semantic similarity method is the introduction of IC contribution factor into the semantic value calculation to take into account both the hierarchical structures of DAGs and the disease specificities. Experiments of IDSSIM and its comparison with three state-of-the-art models, i.e., LNCSIM1, LNCSIM2, and ILNCSIM, were performed on both lncRNADisease database and MNDR database by using evaluation measures of receiver operating characteristic (ROC) curves and area under the curve (AUC) values. Results demonstrated that IDSSIM is superior to compared models, and can improve accuracy of disease semantic similarity effectively, leading to increase the association prediction ability of our model. Besides, case studies of breast cancer and adenocarcinoma were also adopted. Results showed that most of potential disease-associated lncRNAs predicted by IDSSIM can be confirmed by databases and literatures, implying that IDSSIM can serve as a promising tool for predicting lncRNA functions, identifying potential lncRNA-disease associations, and pre-screening candidate lncRNAs to perform biological experiments.

## Methods

### Human lncRNA-disease associations

Two matrices that contain human lncRNA-disease associations were collected for the calculation of lncRNA functional similarities. The first matrix came from the 2017 version of lncRNADisease database [18] (http://www.cuilab.cn/lncrnadisease) in October, 2019. There were in total 116 lncRNAs that were collected according to the reference [19]. After performing quality control to exclude lncRNAs unrecorded in lncRNADisease database and diseases with irregular names or lack of Medical Subject Headings (MeSH) tree numbers, 157 diseases, 82 lncRNAs and 701 associations were retained. The second matrix was downloaded from the Mammalian ncRNA-disease repository (MNDR) database [20] (http://www.rna-society.org/mndr/index.html) in October, 2019. After the same quality control, we collected lncRNA-disease associations with 89 diseases, 190 lncRNAs and 1680 associations. In these two matrices, each row represents an lncRNA and each column represents a disease. If an lncRNA associated with a disease, its corresponding element of matrix is set to 1, otherwise, 0.

### Disease semantic similarity

Disease semantic similarity between two diseases can be calculated using their DAGs, which were constructed by mapping their disease names into MeSH descriptors. MeSH descriptors were obtained from the National Library of Medicine [21] (http://www.nlm.nih.Gov/), and the disease category of which was used here. For a disease *A*, its DAG can be denoted as *DAG*_*A*_ = {*T*_*A*_, *E*_*A*_}, where *T*_*A*_ is the set of ancestor nodes of *A* including itself, and *E*_*A*_ is the set of all edges in the DAG. The disease term *t* ∈ *T*_*A*_ in *DAG*_*A*_ has semantic contribution to the disease *A*, which was defined as semantic value ${SV}_A^1(t)$ of *t* to the disease *A*, and can be calculated in LNCSIM1 [16] by the following formula,

$$ {SV}_A^1(t)=\left\{\begin{array}{cc}1& t=A\\ {}\max \left(\Delta \times {SV}_A^1\left({t}^{\prime}\right)\left|{t}^{\prime}\in C(t)\right.\right)& t\ne A\end{array}\right. $$

where *C*(*t*) is the children of *t*, Δ is the semantic contribution factor of edges in *E*_*A*_ that linking *t* and *t*^′^, which was normally set to 0.5 [22].

This formula interprets the DAG in a quantitative way under the assumption of disease terms at the same layer of *DAG*_*A*_ having the same semantic contribution to the disease *A*. However, this assumption is sometimes problematic. For example, the disease term *t*_1_ and *t*_2_ are at the same layer of *DAG*_*A*_, but compared with *t*_2_, *t*_1_ is a rarer disease and appears in less DAGs. In this case, the conclusion of *t*_1_ being the more specific disease term than *t*_2_ in *DAG*_*A*_ and therefore ${SV}_A^1\left({t}_1\right)$ being higher than ${SV}_A^1\left({t}_2\right)$ seems more reasonable than the assumption of LNCSIM1.

To consider this situation, LNCSIM2 used another formula to calculate the contribution of disease term *t* ∈ *T*_*A*_ in *DAG*_*A*_ to the semantic value of disease *A*,

$$ {SV}_A^2(t)=-\log \frac{Dags(t)}{D} $$

where *D* is the number of diseases in the MeSH, and *Dags*(*t*) is the number of DAGs including *t*. This IC strategy helps to retain the disease specificity, and performs well while several diseases with significantly different DAG-frequencies appear at the same layer of a DAG. However, its accuracy depends on the information integrity of DAGs and easily suffers from the information bias in DAGs.

In the IDSSIM model, we leveraged the advantages of both LNCSIM1 and LNCSIM2, and defined the contribution of disease term *t* ∈ *T*_*A*_ in *DAG*_*A*_ to the semantic value of disease *A* as,

$$ {SV}_A^3(t)=\left\{\begin{array}{cc}1& t=A\\ {}\max \left(\left(\Delta +{P}_t\right)\times {SV}_A^3\left({t}^{\prime}\right)\left|{t}^{\prime}\in C(t)\right.\right)& t\ne A\end{array}\right. $$

where *P*_*t*_ is the IC contribution factor, and defined as,

$$ {P}_t=\frac{\underset{k\in K}{\max}\left( Dags(k)\right)- Dags(t)}{D} $$

where *K* is the set of all diseases in MeSH. It should be noted that for the disease *t*, its *P*_*t*_ value change with the continuously updated version of MeSH.

Then, the semantic value of disease *A* in IDSSIM was calculated in the same way as described in LNCSIM1, that is, it is the summation of contributions of all disease terms in *DAG*_*A*_ to the disease *A*,

$$ SV(A)=\sum \limits_{t\in {T}_A}{SV}_A^3(t) $$

Furthermore, the disease semantic similarity between two diseases *A* and *B* was defined in the similar way as LNCSIM1 based on their shared disease terms in DAGs,

$$ DSS\left(A,B\right)=\frac{\sum \limits_{t\in {T}_A\cap {T}_B}\left({SV}_A^3(t)+{SV}_B^3(t)\right)}{SV(A)+ SV(B)} $$

**Fig. 1:**
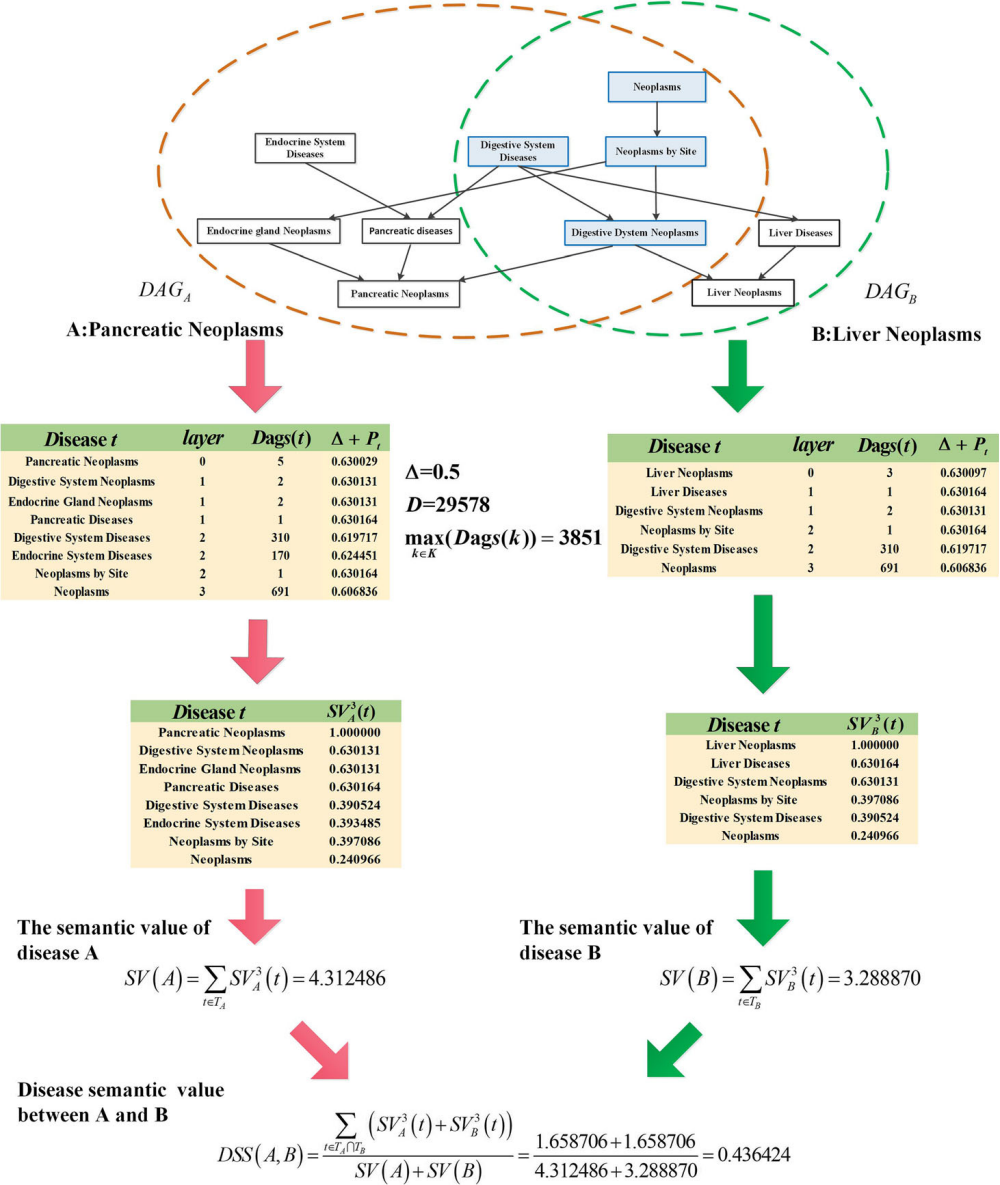
An example of calculating the disease semantic similarity in IDSSIM

To understand the calculation process of the disease semantic similarity more clearly, an example was given in Fig. 1. First, DAGs of two diseases, i.e., Pancreatic Neoplasms and Liver Neoplasms, were constructed by using MeSH descriptors. It is seen that DAG of Pancreatic Neoplasms has 4 layers and 8 disease terms, and DAG of Liver Neoplasms has 4 layers and 6 disease terms, among which, 4 disease terms are shared by these two diseases. Second, *D*, *Dags*(*t*), and $\underset{k\in K}{\max}\left( Dags(k)\right)$ were calculated by using all disease DAGs, and the semantic contribution factor Δ was also set to 0.5 [16, 22]. We can see that disease terms in the same layer have different contribution factor Δ + *P*_*t*_, therefore yielding different semantic contributions ${SV}_A^3(t)$ to the disease in each DAG. Third, semantic values of these two diseases and their disease semantic similarity were calculated using above formulas. As we can see from the example, the IDSSIM model takes into account both the hierarchical structures of DAGs and the disease specificities.

### LncRNA functional similarity

**Fig. 2:**
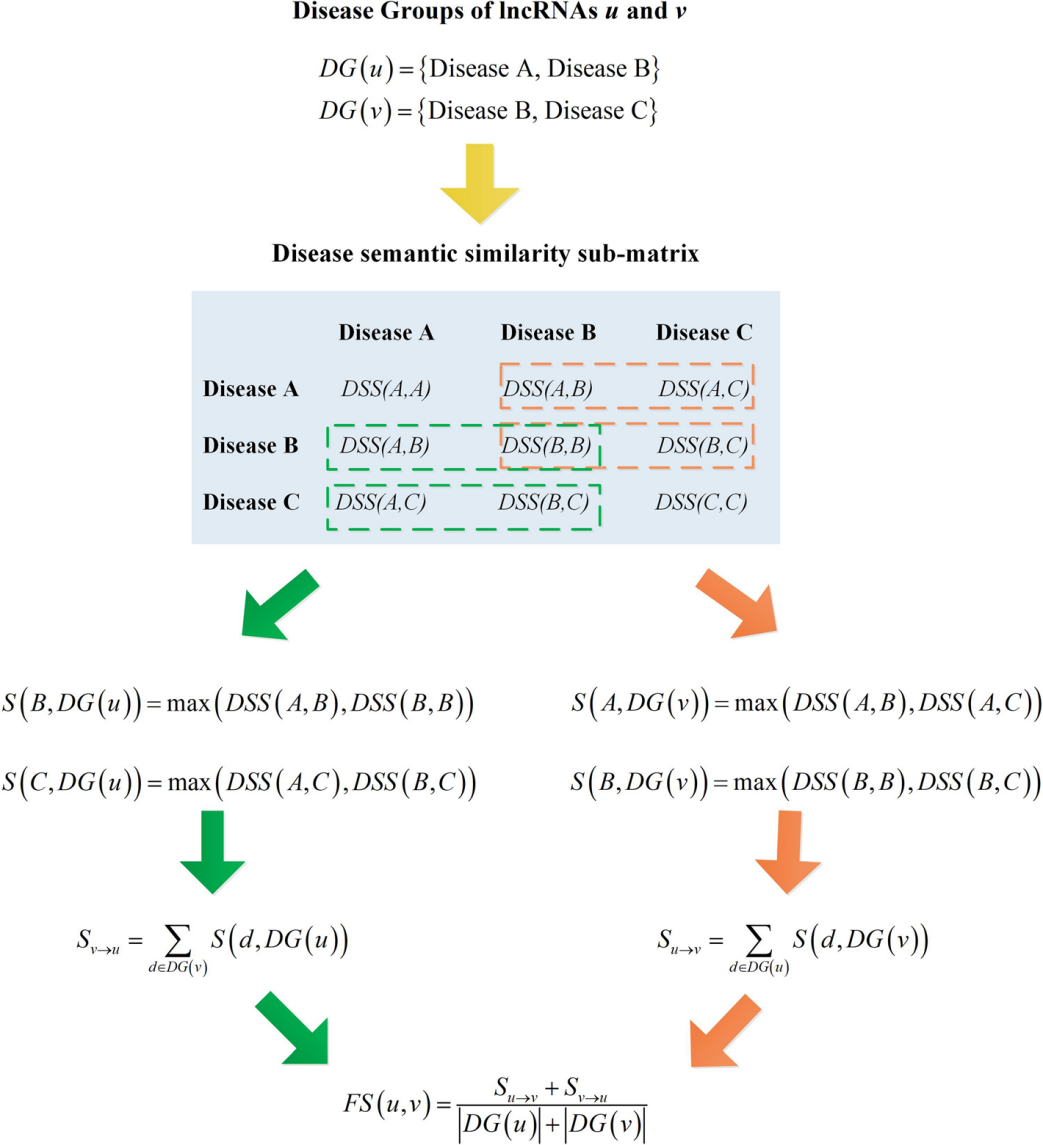
An example of calculating the lncRNA functional similarity in IDSSIM

In the IDSSIM model, the lncRNA functional similarity was calculated in the same way as described in the references [11, 13, 16]. In this paper, an example was given to explain the calculation process, as shown in Fig. 2.

Suppose *DG*(*u*) and *DG*(*v*) are disease groups of lncRNAs *u* and *v* respectively, which were collected from the matrix of human lncRNA-disease associations, the lncRNA functional similarity between *u* and *v* can be calculated using semantic similarities of diseases appearing in *DG*(*u*) and *DG*(*v*). More specifically, at first, the disease semantic similarity sub-matrix was constructed, where both row and column represent diseases that appears in *DG*(*u*) ∪ *DG*(*v*), and each element is the disease semantic similarity between the corresponding diseases. Then, the similarity between a disease of one disease group and another disease group is defined as,

$$ S\left({d}_u, DG(v)\right)=\underset{d\in DG(v)}{\max}\left( DSS\left({d}_u,d\right)\right) $$

$$ S\left({d}_v, DG(u)\right)=\underset{d\in DG(u)}{\max}\left( DSS\left({d}_v,d\right)\right) $$

where *d*_*u*_ and *d*_*v*_ represent one disease in *DG*(*u*) and *DG*(*v*), respectively. Next, the similarities of two disease groups to each other were defined as,

$$ {S}_{u\to v}=\sum \limits_{d\in DG(u)}S\left(d, DG(v)\right) $$

$$ {S}_{v\to u}=\sum \limits_{d\in DG(v)}S\left(d, DG(u)\right) $$

Finally, the lncRNA functional similarity between *u* and *v* was defined as,

$$ FS\left(u,v\right)=\frac{S_{u\to v}+{S}_{v\to u}}{\left| DG(u)\right|+\left| DG(v)\right|} $$

where |⋅| denotes the number of diseases in the corresponding disease group.

## Results and discussion

### Performance evaluation

In order to evaluate the performance of IDSSIM, we compared it with three state-of-the-art models, i.e., LNCSIM1, LNCSIM2, and ILNCSIM, on both lncRNADisease database and MNDR database by using evaluation measures of ROC curves and AUC values that generated by a five-fold cross validation strategy [13].

Specifically, for each database, the original matrix of human lncRNA-disease associations was randomly divided into five groups, scores of one of which were changed into 0 and others remained unchanged. These five changed association matrices, as well as results of each compared model, i.e., disease semantic similarity matrix and lncRNA functional similarity matrix, were applied to an association prediction method WKNKN [23] in turn to get five predicted matrices of human lncRNA-disease associations. WKNKN was used here since it was recently proposed and claimed to facilitate association prediction and its package is available online. For the changed group in the original matrix of human lncRNA-disease associations, associations with their scores being equal to 1 were considered as observed positives, otherwise, observed negatives. For the changed group in each predicted matrix of human lncRNA-disease associations, associations with their scores being higher than a threshold were considered as predicted positives, otherwise, predicted negatives, where the threshold was set to predicted scores in the changed group with the descending order in turn. Therefore, for each predicted matrix of human lncRNA-disease associations, their true positive rates (TPR) and false positive rates (FPR) can be obtained with different thresholds. In order to reduce the error caused by random grouping, the five-fold cross validation was repeated 10 times for each compared model, and the average values of TPR and FPR were used to draw ROC curve and calculate AUC value.

**Fig. 3:**
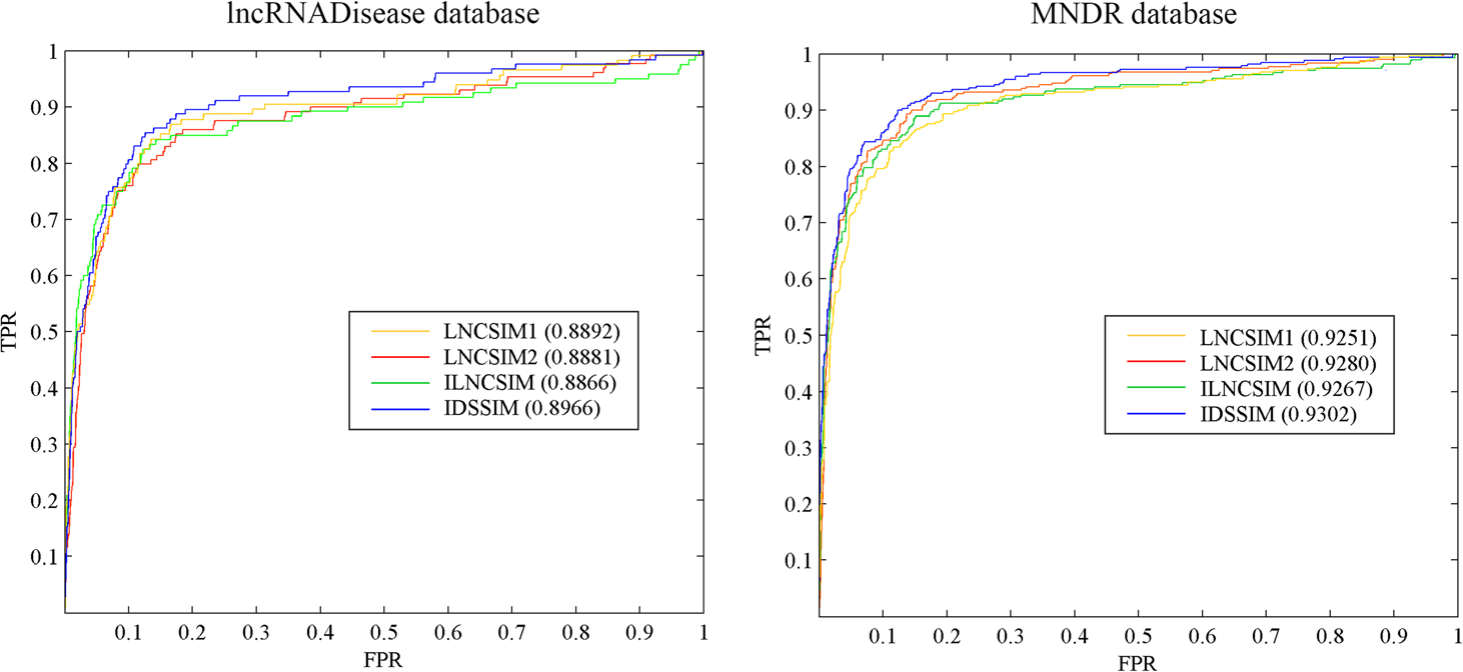
ROC curves and AUC values of compared models on lncRNADisease database and MNDR database

ROC curves and AUC values of compared models on lncRNADisease database and MNDR database were shown in Fig. 3. It is seen that in terms of ROC curves and AUC values, IDSSIM performed best among all compared models on these two databases. For the lncRNADisease database, the AUC value of IDSSIM was 0.8966, and 0.74, 0.85, 1.00% higher than AUC values of LNCSIM1, LNCSIM2, ILNCSIM, respectively. Similarly, for the MNDR database, the AUC value of IDSSIM was 0.9302, has increased by 0.51, 0.22 and 0.35% than those of LNCSIM1, LNCSIM2, ILNCSIM, respectively. These experimental results demonstrated that IDSSIM can provide more accurate disease semantic similarity matrix and lncRNA functional similarity matrix. Therefore, based on these two matrices, performance of the association prediction method, such as WKNKN, can be further improved.

**Fig. 4:**
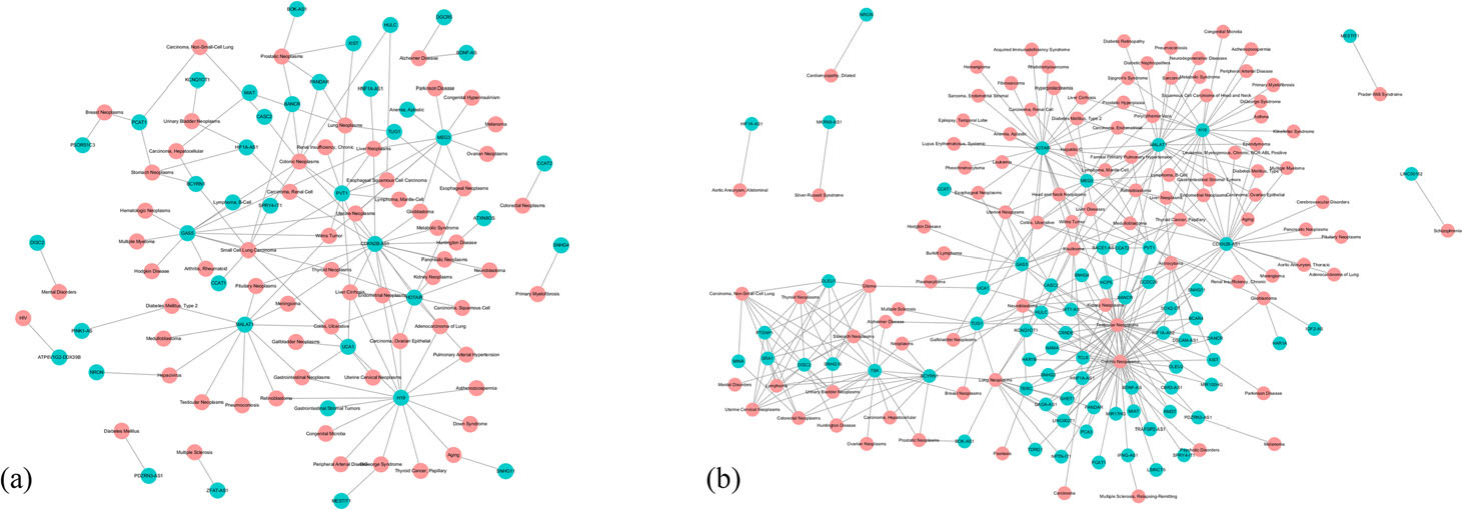
The significant potential lncRNA-disease association networks captured by IDSSIM

We applied two similarity matrices that generated by IDSSIM, namely, the disease semantic similarity matrix and the lncRNA functional similarity matrix, as well as their corresponding downloaded matrix of human lncRNA-disease associations coming from either lncRNADisease database or MNDR database, to the association prediction method WKNKN [23] to get two predicted matrices of human lncRNA-disease associations. In these two predicted matrices, several potential lncRNA-disease associations were identified, which might be useful for uncovering underlying genetic mechanisms of diseases though they need further bioinformatics studies and biological experiment confirmation. In Fig. 4, the significant potential lncRNA-disease associations captured by IDSSIM were shown as networks. In each network, blue and red nodes represent lncRNAs and diseases respectively, and each edge linking an lncRNA and a disease represents the captured significant potential lncRNA-disease association, score of which is higher than a threshold *m*(*LDA*) + 2 ⋅ *sd*(*LDA*), where *LDA* denotes scores of all potential lncRNA-disease associations that captured by IDSSIM, *m*(⋅) and *sd*(⋅) are the mean and the standard deviation of them. We believed that these two networks can provide important clues for the exploration of causative biomarkers of diseases.

### Case studies

Based on the predicted matrix of human lncRNA-disease associations in the lncRNADisease database, another evaluation method of case studies, which is a routine method and has been widely adopted by association prediction models [23, 24], was used to validate the effectiveness of IDSSIM. Two diseases, i.e., breast cancer and adenocarcinoma, were taken as cases in the study. For each disease, top 20 predicted potential lncRNAs were recorded, as shown in Table 1 and Table 2 respectively. In the tables, lncRNAs were examined one by one to confirm whether it associates with the disease using the lncRNADisease (v2.0) database [25], Lnc2Cancer database [26] and recently published literatures.

**Table 1:** Top 20 predicted potential lncRNAs associated with breast cancer

Rank	lncRNA	Evidence
**1**	**PCAT1**	**[30]**
2	PSORS1C3	Unconfirmed
**3**	**MIAT**	**LNC2Cancer**
4	HIF1A-AS1	Unconfirmed
**5**	**BANCR**	**LNC2Cancer**
**6**	**CASC2**	**LNC2Cancer**
**7**	**GHET1**	**LNC2Cancer**
**8**	**PTENP1**	**lncRNADisease(v2.0)**
9	7SK	Unconfirmed
10	DNM3OS	Unconfirmed
**11**	**HULC**	**LNC2Cancer**
12	NPTN-IT1	Unconfirmed
13	MINA	Unconfirmed
**14**	**SNHG3**	**[31]**
15	SNHG4	Unconfirmed
**16**	**MIR100HG**	**[32]**
**17**	**CRNDE**	**LNC2Cancer/lncRNADisease(v2.0)**
**18**	**WRAP53**	**[33]**
**19**	**SNHG16**	**LNC2Cancer/lncRNADisease(v2.0)**
20	BOK-AS1	Unconfirmed

**Table 2:** Top 20 predicted potential lncRNAs associated with adenocarcinoma

Rank	lncRNA	Evidence
**1**	**GAS5**	**[38]**
**2**	**HOTAIR**	**[39]**
**3**	**MALAT1**	**[40]**
**4**	**MEG3**	**[41]**
**5**	**H19**	**[42]**
**6**	**CCAT1**	**[43]**
7	HULC	Unconfirmed
8	NAMA	Unconfirmed
9	MIAT	Unconfirmed
10	WT1-AS	Unconfirmed
**11**	**PANDAR**	**[44]**
12	PTENP1	Unconfirmed
**13**	**PVT1**	**[45]**
14	TUG1	Unconfirmed
**15**	**UCA1**	**[46]**
16	BANCR	Unconfirmed
17	CBR3-AS1	Unconfirmed
**18**	**CCAT2**	**[47]**
19	CDKN2B-AS1	Unconfirmed
**20**	**DANCR**	**[48]**

Breast cancer is one of the most common malignant tumors which threaten the health of women, accounting for about 500,000 deaths per year worldwide [27]. Recent advances have suggest that dysregulations of lncRNAs are associated with breast cancer [28, 29]. Besides known associations between lncRNAs and breast cancer in the lncRNADisease database, we further predicted 20 potential lncRNAs in Table 1 that might be involved with breast cancer. Among them, 8 lncRNAs have been confirmed by lncRNADisease (v2.0) database and Lnc2Cancer database, and 4 lncRNAs were reported by literatures to be implicated in breast cancer. Sarrafzadeh et al. demonstrated that significant up-regulation of PCAT1 has only been detected in a fraction of breast cancers and concluded that PCAT1 is possibly involved in the pathogenesis of fraction of breast cancers [30]. Ma et al. declared that SNHG3 promotes cell proliferation and invasion through the miR-384/hepatoma-derived growth factor axis in breast cancer [31]. Wang et al. identified MIR100HG as a pro-oncogene for triple-negative breast cancer progression that promotes cell proliferation through triplex formation with p27 loci [32]. Silwal-Pandit et al. showed that the sub-cellular localization of the WRAP53 protein has a significant impact on breast cancer survival, and thus has a potential as a clinical marker in diagnostics and treatment [33].

Adenocarcinoma is a type of malignant tumors, and appears in many human organs, for example, lung [34], prostate [35], stomach [36], colon [37] and so on. Among top 20 predicted potential lncRNAs in Tables 2, 11 lncRNAs were reported to be associated with adenocarcinoma in literatures. Dong et al. showed that GAS5 is significantly downregulated in lung adenocarcinoma tissues, and may represent a potential biomarker for the diagnosis of lung adenocarcinoma [38]. Lee et al. found that HOTAIR was involved in inhibition of apoptosis and promoted invasiveness, supporting a role for HOTAIR in carcinogenesis and invasion of gastric adenocarcinoma [39]. Tano et al. suggested that MALAT1 enhances cell motility of lung adenocarcinoma cells by influencing the expression of motility-related genes [40]. Li et al. confirmed that MEG3 plays a promoting role in the proliferation, invasion, and angiogenesis of lung adenocarcinoma cells through the AKT pathway [41]. Liu et al. reasoned that H19 promotes viability and epithelial-mesenchymal transition of lung adenocarcinoma cells by targeting miR-29b-3p and modifying STAT3 [42]. Lin et al. concluded that overexpression of CCAT1 promotes metastasis via epithelial-to-mesenchymal transition in lung adenocarcinoma [43]. Jiang et al. found that an increased expression of PANDAR promotes cell proliferation and inhibits cell apoptosis in pancreatic ductal adenocarcinoma [44]. Xu et al. provided strong evidence that PVT1 confers an aggressive phenotype to esophageal adenocarcinoma [45]. Liu et al. suggested that UCA1 axis plays a crucial role in progression of pancreatic ductal adenocarcinoma and may serve as a target for new therapies [46]. Hu et al. showed that CCAT2 may act as a competitive endogenous RNA to regulate FOXC1 expression by competitively binding miR-23b-5p in lung adenocarcinoma [47]. Lu et al. suggested that DANCR might be an oncogenic lncRNA that regulates mTOR expression through directly binding to miR-496, and therefore may be regarded as a biomarker or therapeutic target for lung adenocarcinoma [48].

Though future studies are needed to confirm above findings, according to case studies, we believed that IDSSIM is a promising model for lncRNA function prediction, and the time and cost could be significantly reduced while performing biological experiments based on clues that provided by IDSSIM.

**Fig. 5:**
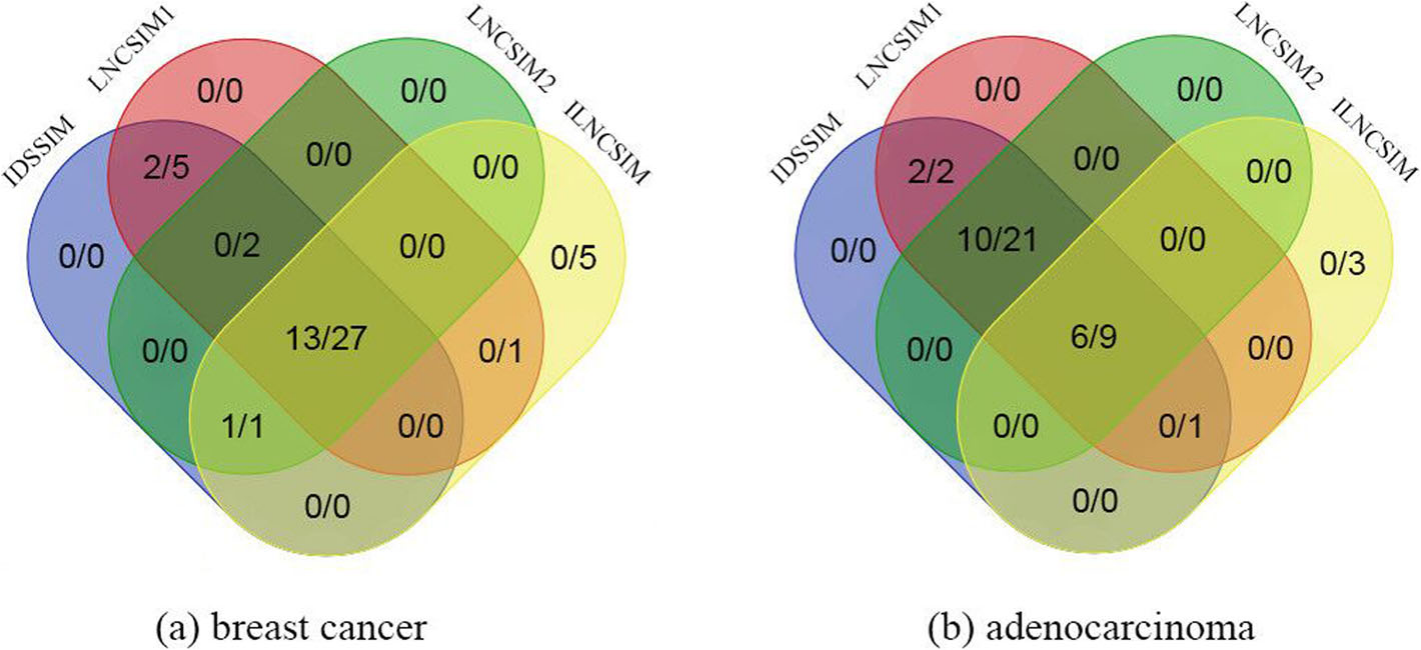
Venn diagrams of four compared models on breast cancer and adenocarcinoma

In order to further validate the effectiveness of IDSSIM, Venn diagrams of four compared models were illustrated in Fig. 5, each element of which can be written as |*L*_*con*_|/|*L*_*all*_|, where *L*_*all*_ represents potential disease-associated lncRNAs that predicted by all corresponding models, *L*_*con*_ represents those lncRNAs in *L*_*all*_ that can be confirmed to associated with the disease by databases and literatures, and |⋅| denotes the number of *L*_*all*_ or *L*_*con*_. It is seen that the combination of IDSSIM and WKNKN can predict more confirmed disease-associated lncRNAs than other combinations of compared models and WKNKN. For breast cancer, IDSSIM predicted 35 potential disease-associated lncRNAs in total and 16 out of which have been confirmed. These ratios of LNCSIM1, LNCSIM2, and ILNCSIM were 15/35, 14/30, and 14/34 respectively. Similarly, for adenocarcinoma, these ratios of IDSSIM, LNCSIM1, LNCSIM2, and ILNCSIM were 18/33, 18/33, 16/30, and 6/13 respectively.

## Conclusions

LncRNA functional similarity calculation model plays an important role in predicting lncRNA functions and identifying potential lncRNA-disease associations. In this paper, we proposed an lncRNA functional similarity calculation model, IDSSIM for short, based on an improved disease semantic similarity method, highlight of which is the introduction of IC contribution factor into the semantic value calculation to take into account both the hierarchical structures of DAGs and the disease specificities. To evaluate the performance of IDSSIM, comparison experiments with three state-of-the-art models LNCSIM1, LNCSIM2, and ILNCSIM, were performed on both lncRNADisease database and MNDR database by using evaluation measures of ROC curves and AUC values. Results demonstrated that IDSSIM is superior to compared models, and can improve accuracy of disease semantic similarity effectively, leading to increase the association prediction ability of our model. In addition, case studies of breast cancer and adenocarcinoma were also adopted. Results showed that most of potential disease-associated lncRNAs predicted by IDSSIM can be confirmed by databases and literatures, implying that IDSSIM can serve as a promising tool for predicting lncRNA functions, identifying potential lncRNA-disease associations, and pre-screening candidate lncRNAs to perform biological experiments.

However, IDSSIM still has several limitations, which inspire us to continue working in the future. Firstly, the information biases of diseases and/or lncRNAs in databases which usually caused by their research heat sometimes lead to inaccurate lncRNA-disease association scores. Secondly, the priori knowledge of lncRNAs, as well as their interactions with other biomolecules, should be considered together in IDSSIM to further improve its prediction accuracy. Thirdly, software package or web application of IDSSIM should be provided later.

## Data Availability

The IDSSIM code and experimental data, including the matrices of the human lncRNA-disease associations that comes from the lncRNADisease database and the MNDR database respectively, two corresponding disease semantic similarity matrices, two corresponding lncRNA functional similarity matrices, and two corresponding matrices of the human lncRNA-disease associations that predicted by WKNKN, are available online at https://github.com/CDMB-lab/IDSSIM.

## Abbreviations

LncRNA: Long non-coding RNA
NcRNAs: Non-coding RNAs
GO: Gene Ontology
MeSH: Medical Subject Headings
MNDR: Mammalian NcRNA-Disease Repository
DAGs: Directed Acyclic Graphs
IC: Information Content
AUC: Area Under the Curve
WKNKN: Weighted K Nearest Known Neighbors
TPR: True Positive Rates
FPR: False Positive Rates
ROC: Receiver Operating Characteristic
